# Long term follow-up study to evaluate immunogenicity and safety of a single dose of live attenuated hepatitis a vaccine in children

**DOI:** 10.4161/21645515.2014.979646

**Published:** 2015-05-27

**Authors:** Monjori Mitra, Nitin Shah, MMA Faridi, Apurba Ghosh, VS Sankaranarayanan, Anju Aggarwal, Suparna Chatterjee, Nisha Bhattacharyya, Ganesh Kadhe, Gaurav Vishnoi, Amey Mane

**Affiliations:** 1 Institute of Child Health; Kolkata, India; 2 Department of Pediatrics; Lion's Tarachand Bapa Hospital; Sion West, Mumbai, India; 3 Department of Pediatrics; University College of Medical Sciences; GTB Hospital, Dilshad Garden; Delhi, India; 4 Kanchi Kamakoti Childs Trust Hospital; Chennai, India; 5 Department of Pediatrics; University College of Medical Sciences; GTB Hospital; Delhi, India; 6 Deptartment of Pharmacology; Institute of Postgraduate Medical Education & Research; Kolkata, India; 7 Medical Affairs; Wockhardt Limited, Wockhardt Towers; Bandra–Kurla Complex; Bandra–East, Mumbai

**Keywords:** antibody, children, hepatitis A, immunization, live attenuated vaccine

## Abstract

Worldwide, viral hepatitis continues to be a cause of considerable morbidity and mortality. Mass immunization with a single dose of live attenuated HAV has been shown to significantly reduce disease burden in the community. This was a phase IV, 5-year follow up study carried out at 4 centers (Kolkata, Delhi, Mumbai and Chennai) across India. The subjects with antibody titer <20 mIU/mL at baseline were evaluated for long term immunogenicity. Of the 503 subjects enrolled, 349 subjects were baseline seronegative with an anti-HAV antibody titer <20 mIU/mL. Overall, 343 subjects could be followed up at some point of time during this 5 y post vaccination period. In the last year (60 months) of follow-up, 108 subjects (97.3%) of 111 subjects (who came for follow-up at the end of 5 y) had a protective antibody titer (anti-HAV antibody titer >20 mIU/mL). The seroconversion rates considering seroprotection levels of anti-HAV antibody titer >20 mIU/mL, following vaccination starting from 6 weeks, 6 months, 12 months, 24 months, 36 months, 48 months and 60 months were 95.1%, 97.9%, 98.3%, 96.2%, 97.8%, 92.6% and 97.3%, respectively. The geometric mean concentration (GMC) over the years increased from 64.9 mIU/mL at 6 weeks to 38.1 mIU/mL and 135.2 mIU/mL at 6 months and 12 months, respectively and was maintained at 127.1 mIU/mL at 60 months. In conclusion, the result of this 5-year follow up study showed that the single dose of live attenuated vaccine is well tolerated and provides long-term immunogenicity in healthy Indian children.

## Abbreviations

GMC: Geometric mean concentration
WHO: World Health Organization
CDC: Center for Disease Control and Prevention
CIs: Confidence intervals
Aes: Adverse events

## Introduction

Vaccination is an effective measure to provide immunization against hepatitis A virus (HAV) infection and reduce its incidence.^1-3^ World Health Organization (WHO) recommends routine immunization programs in countries where a large proportion of the adult population is susceptible to HAV.^4^ Large scale immunization programs are likely to be a cost-effective public health tool to control HAV infection in developing countries.^4^ According to the US Centers for Disease Control and Prevention (CDC), early childhood vaccination would help in the prevention of HAV infection in children and infants that account for at least one-third of the HAV cases. Immunization might also eliminate the major source of infection for other children by cross contamination; and ultimately prevent infection in the older age groups as the immunity provided by HAV vaccines is long-lasting.^5^

Two types of vaccines (live attenuated vaccine and inactivated vaccine) are available in India for vaccination against HAV infection. Live attenuated hepatitis A virus vaccines (H2 and L-A-1 strains) have been developed, manufactured and licensed in China.^6^ The only other country where live attenuated vaccine is licensed is India (H2 strain).^6^

Attenuation in live attenuated vaccines is attained through multiple cell culture passages and subsequent propagation in human diploid embryonic lung fibroblast cells.^7^ Live attenuated vaccines contain treated (altered to a non-pathogenic form) and weakened (attenuated) form of virus that causes infection similar to the natural infection to a limited extent, necessary only to stimulate the immune response in an individual.^8^

According to the recommendations of WHO, it is necessary to confirm the long-term efficacy and immunogenicity of a single dose of live attenuated vaccines through regular monitoring and evaluation plans.^9^ A previous study from a single center in Pune, India showed a good immunogenic response (95.8%) and tolerability profile with vaccine after 30 months post vaccination.^10^ The present study was a multicentre, follow-up post marketing study of live attenuated vaccine (Biovac-A™) to evaluate long-term persistence (up to 5 y) of the protective anti HAV antibody titer in subjects who were vaccinated with a single dose of this vaccine.

## Results

The total number of subjects enrolled was 503, who were vaccinated and monitored for adverse events following immunisation (AEFI) and safety. The subjects with an antibody titer <20 mIU/mL at baseline were evaluated for long term immunogenicity. Over 5 y, 343 subjects came for follow-up and were bled at least 2 times following vaccination. At 5 y post vaccination visit, 111 subjects came for follow-up.

**Figure 1.**
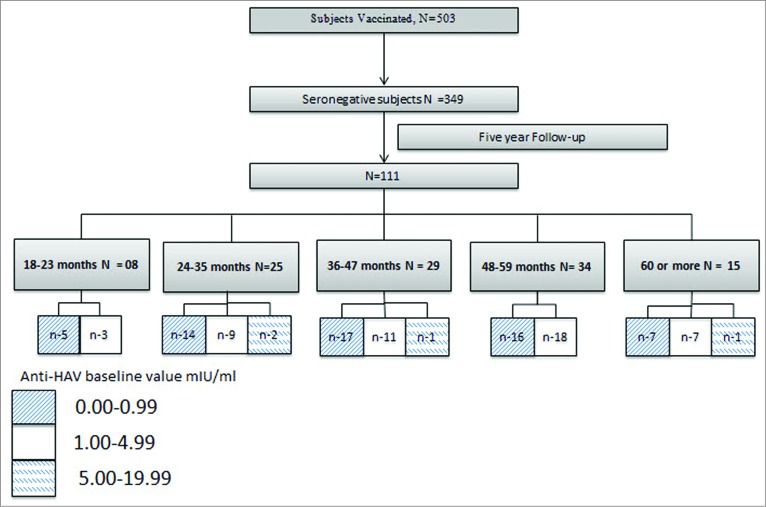
Distribution of enrolment age group with the baseline anti HAV antibody titer.

Of the 503 subjects enrolled, 349 subjects were baseline seronegative with anti-HAV antibody titer <20 mIU/mL (**Fig. 1**). Of these 349 subjects, only 6 subjects never came for a follow-up visit. The number of children observed at each clinical review at specific interval were 334 (6 weeks), 290 (6 months), 186 (12 months), 133 (24 months), 135 (36 months), 102 (48 months), and 111 (60 months).

At the various time points of the follow-up visit, it was observed that the subjects who seroconverted after 6 weeks had continued to attain the seroprotective level and 2.7% subjects who had an anti-HAV antibody titer between 10–20 mIU/mL at 5 y had a low anti-HAV antibody titer all through. None of these patients had any breakthrough infection during the study period.

Overall, 95.1% of the subjects developed detectable anti-HAV antibody titer >20 mIU/mL (GMC 64.9 mIU/mL), after 6 weeks of a single dose vaccine administration. After 6 months, 12 months and 60 months of vaccination, 97.9% (GMC 138.1 mIU/mL), 98.3% (GMC 135.2 mIU/mL) and 97.3% (GMC 127.1 mIU/mL) of the subjects attained anti-HAV antibody titer >20 mIU/mL, respectively. The seroconversion rate following single dose of live attenuated hepatitis A vaccine was calculated at cut off value of >20 mIU/ml and the GMC was calculated by excluding the subjects who had an anti-HAV antibody titer >10,000 mIU/ml. All the subjects with titer values less than the seroprotective levels were also included in the analysis (**Table 1**).

**Table 1.** Percentage seroconversion following single dose of live attenuated hepatitis A vaccine (at cut off value of >20mIU/ml) and GMC (excluding subjects antibody titer >10.000 mIU/mL)

			Excluding antibody titer >10,000 mIU/mL	
Time	Subjects	Seropositivity rate (%) (at cut off value of >20mIU/ml)	GMC	95% CI
6 wk	334	95.1	64.9	58.4–72.2
6 mon	290	97.9	138.1	119.2–147.1
12 mon	186	98.3	135.2	110.4–147.7
24 mon	133	96.2	124.6	96.2–152.9
36 mon	135	97.8	137.6	104.5–149.2
48 mon	102	92.6	127.4	91.3–155.1
60 mon	111	97.3	127.1	70.1–156.3

If all the subjects are considered including the subjects with anti-HAV antibody titer >10,000 mIU/mL (subclinical infection due to natural infection), the GMC ranged from 81.0 mIU/mL at 6 weeks post vaccination to 148.9 mIU/mL at 60 months (for anti-HAV antibody cut-off titers for seroprotection >20 mIU/mL), respectively showing a stability with respect to the GMC values observed in the past 5 y.

**Figure 2.**
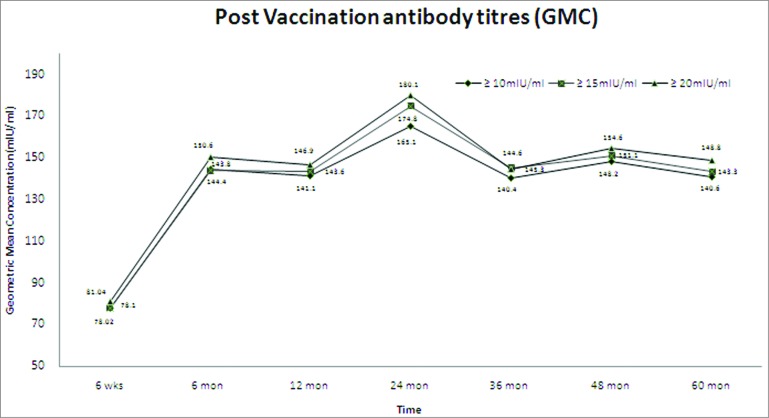
Geometric mean concentration (GMC) for different age groups for subjects with baseline HAV antibody titer <20 mIU/mL including all tested subjects.

It was observed that the subjects who did not seroconvert initially after 6 weeks, had attained seroprotected titer based on the different cut-off values (>10 mIU/mL, >15 mIU/mL and >20 mIU/mL) at some point of the follow-up visits. Of these subjects, 49 came for all the visits in each year and had a GMC of 116.6 mIU/mL (91.7 mIU/mL–148.4 mIU/mL). The GMC for different age groups for the subjects with baseline HAV antibody titer <20 mIU/mL is presented in **Figure 2**.

**Figure 3.**
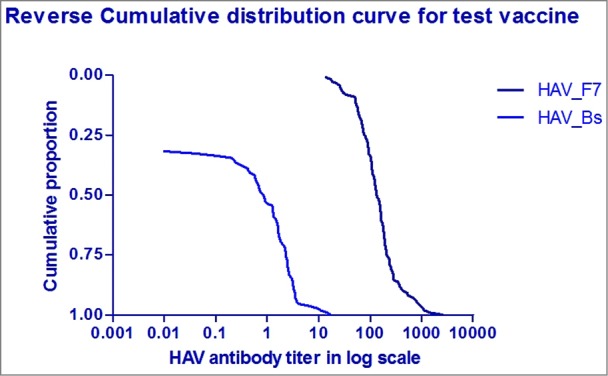
Reverse cumulative distribution curve of anti-HAV antibody titer at baseline and after 60 months post vaccination.

No reports of hepatitis like illnesses were recorded during the follow-up visits in any of the subjects since vaccination. There has been a persistence of immunogenicity in all the children who had come for the follow-up visit after 5 y with a significantly high titer value. The reverse distribution curve shows the immune response at the 5 y was above the baseline titer level (**Fig. 3**). In 2% to 7% subjects there was a drop in titer value <20 mIU/mL, but none had any clinical history of jaundice. Similarly, the subjects with a high antibody titer >10,000 mIU/mL did not have any clinical history of hepatitis A. However, these subjects could be considered as having probable subclinical infection, thus substantiating the anamnestic response. Among the 349 subjects, 23 subjects had a response >5000 mIU/mL and one subject had a titer value >10,000 mIU/mL twice in subsequent years but clinically had no history of icterus.

## Discussion

The present 5- year follow-up study showed that the live attenuated HAV vaccine provides long-term immunity with a single dose of the vaccine. A total of 97.3% patients seroconverted with a GMC of 127.1 mIU/mL (102.3 mIU/mL–158.1 mIU/mL) at the end of 60 months. These results were consistent with the results of the previous studies conducted with the same vaccine in China.

Worldwide, viral hepatitis continues to be a cause of considerable morbidity and mortality. Approximately, 1.4 million cases of hepatitis A are reported every year.^9,11^ The improvement in the socioeconomic and sanitation conditions in India during the past 2 decades has led to an epidemiological shift in the prevalence of HAV infection with adults at a higher risk of HAV infection than the children.^9,11-13^

In China, live HAV vaccines have been used for over 20 y and have shown remarkable safety, immunogenicity, and long-term protection to millions of subjects.^8^ A reduced incidence of Hepatitis A in the Chinese regions was observed after mass and routine public immunization programs of the vaccine.^1,14-16^ Previous studies with live attenuated HAV vaccines have reported good immunogenicity. Zuhang et al. in their study observed and confirmed the persistence and long-term efficacy of live attenuated HAV vaccine after mass immunization. The seroconversion observed in follow-up assay for 2 months and 10 y after inoculation was 98.6% and 80.2% respectively.^1^ Mao et al. in their study showed satisfactory protective efficacy of H2-strain vaccine after the 4-year follow up of the schoolchildren vaccinated with live attenuated HAV vaccine. The results of this study demonstrated that the vaccine is effective with the ability of achieving 100% protection rate.^14^ In 8-year follow up study by Wang et al., 72% (61/85) seropositive subjects were observed after 96 months of immunization (GMC 89.0 mIU/mL) that showed the satisfactory long term immunogenicity provided by a single dose of live attenuated HAV vaccine.^17^

Vacchino et al. in their study observed a sharp reduction in hepatitis A rates (88%) across all age group after the mass vaccination program with a single dose inactivated HAV vaccine in Argentina during 1998–2002.^18^ Pérez et al. reported 100% protective efficacy in children of age 1.5 to 6 y after 6 weeks post vaccination with a single dose inactivated HAV vaccine. ^19^

Ott et al. reported that the protective anti-HAV antibody levels can persist for almost 11 y after a single dose of inactivated hepatitis A vaccine.^20^ In our study, though all the 111 subjects could not be reviewed at various time points but the trend shows that those who had attained (95%) seroprotective titer at 6 weeks had maintained positive titer through out. This should further support the use of a single dose of live attenuated hepatitis A vaccine in intermediate endemic zones prevailing in India.

As compared with the inactivated vaccines, live attenuated vaccines (H2 strain derived) are more genetically stable, more immunogenic in animal studies and non- transmissible by the oral route.^8,21^ The vaccine induces neutralizing antibody and cell-mediated immune response that suggests the possibility of long-term immunity.^1,22^ The revertants of live attenuated HAV vaccine has never been studied among humans. These vaccine are considered to be safe, however there is a rare possibility that an attenuated microbe in the vaccine could revert to a virulent form to cause the disease.^23^ Only 22 cases of reversion to virulence have been documented in over 300 million doses of live attenuated yellow fever (YF- strain 17D) vaccine administered. ^24^

Previous studies have not reported significant AEs related to the HAV vaccines. The most common AEs observed with HAV vaccines are fever, pain, redness, and swelling at the injection site that resolve within few hours or days.^25,26^ In our study, we did not observe any AE post vaccination.

There is no long-term multicentric data in India on persistence of immunogenicity with a single dose of live attenuated hepatitis A vaccine and the impact on the GMC due to prevalence of natural infection causing natural boosting. To our knowledge, the present study is the first long term follow-up study conducted in India for evaluating the immunogenicity and safety of a single dose of live attenuated HAV vaccine in children. The high attrition rate of this multicentric study owing to migratory population and observational nature is a shortcoming. Most of the subjects dropped out due to transfer of the subjects to different geographical location or unwillingness of the child to bleed. No drop out was related to the safety issues. We did not observe any uniform pattern in the follow up visits of the subjects. The subjects visited at different time points for follow up and some of them missed one or 2 visits during the study, The retrospective data shows that most of the subjects could be monitored for a response at least more than 3 times at various time intervals. The trend of GMC and the seroconversion had a rising trend which does mitigate the biasness due to the missing data. None of the subjects who once attained a seroprotectivetiter value had a decline in titer value <20 mIU/mL, but those who did not seroconvert at 6 weeks had a fluctuating titer value at some point of time between (10–20 mIU/mL) at 5 y The subclinical infection >10,000 mIU/mL and the anamnestic response >3000 mIU/mL in the subjects were the reasons for the long-term persistence of antibody through natural boosting effect considering >20 mIU/mL as a positive response. However, it is not possible to completely rule out that the natural exposure to HAV can act or acted as a natural booster among the study participants and resulted in antibody persistence. Future research to recognize the interplay between detectable antibody levels and memory response due to natural infection would be an interesting approach.

## Conclusion

The result of this 5-year follow up study showed that the single dose of live attenuated vaccine is sufficient to provide long term immunogenicity in healthy Indian children between 1–12 y The vaccine is safe and well tolerated. In communities experiencing recurrent epidemics or outbreaks, the use of vaccination with a single dose of live attenuated vaccine seems to be an acceptable cost-effective alternative. According to WHO, a single dose hepatitis A vaccine schedule will be an option for countries introducing universal hepatitis A vaccine into their national immunization schedule.^27^

However, the implementation of routine vaccination of children and/or adolescents that has recently proven to be effective in general HAV epidemiology seems in the long-term, the most reasonable way to get recurrent outbreaks under control in areas where hepatitis A represents a public health problem.

## Materials and Methods

### Subjects and study design

This was an open labeled, study with single dose of live attenuated hepatitis A vaccine to evaluate the long term persistence of immunogenecity and a 5-year follow up study carried out at 4 centers (Kolkata, Delhi, Mumbai and Chennai) acrossIndiabetween 2007 till 2012. Subjects who fulfilled the eligibility criteria were intimated either by post or through the social worker regarding the continuation of the study till 60 months post vaccination. Serum anti HAV antibody titer <20 mIU/ml was taken as susceptible for HAV infection.

In the year 2007, initially a total of 503 subjects were recruited from the out-patient department of the 4 centers and pre immunization blood sample was drawn for measuring base line anti-HAV antibodies and the same were vaccinated with a single dose of live attenuated vaccine (Biovac A™, H2 strain, freeze dried, live vaccine, developed by Zhejiang Pukang Biotechnological Company Ltd, China, imported and marketed in India by Wockhardt Limited). Of the 503 subjects, 349 vaccinated subjects who were HAV susceptible at baseline, were followed-up for the next 5 y (until 5 y time point) with pre-defined annual visits. To assess the persistence of immunogenicity of the live attenuated HAV vaccine, blood samples were collected from subjects and tested for anti-HAV antibody levels at 6 weeks and then at 6, 12, 24, 36, 48 and 60 months after the initial immunization.

During the follow-up visits, participants having anti-HAV titers >10,000 mIU/mL were excluded from the study as the titer value of >10,000 mIU/mL was considered as a subclinical infection and responsible for natural infection giving rise to long-term immunogenicity.

The method used for the quantitative estimation of HAV antibody in this study was Axsym® - HAVAB 2.0 Quantitative assay, based on the principle of Microparticle Enzyme Immunoassay (MEIA), on the kit Abbott Axsym.

### Eligibility criteria

Subjects with a baseline anti-HAV antibody titer of <20 mIU/mL at the beginning of the study were included. Those with a history of clinical jaundice or vaccination were excluded from the study.

### Consent and study approval

Written informed consent was obtained from the parent or guardian of the subjects and the subject prior to enrolment following an explanation of the purpose of the study. The institutional ethics committee of each site approved the trial.

### Statistical analysis

Seroconversion in previously seronegative subjects was defined as the presence of anti-HAV antibody at cut-off levels of 20 mIU/mL (seroprotection). For comparison of geometric mean values and 95% confidence intervals (CIs), and comparisons over different age groups, Student's t-test was used for parametric data. All analyses were 2-tailed with *p* = 0.05 as the cut-off level for statistical significance. Data analysis was carried out by using the SPSS statistical software version 16 (IBM SPSS software, USA).
